# The Dundee "Social Union"

**Published:** 1905-03-18

**Authors:** 


### The Dundee "Social Union."

The late Mr. Stacy Marks, R.A., having completed his admirable painting of a naturalist, standing with a tape in his hand before the skeleton of a bird in a museum, asked a distinguished London physician for a suggestion concerning the title which should be attached to the picture. He was advised to call it "Science is Measurement," and under that name it was given to the world. The profound truth embodied in the phrase has lately been accepted, in a very special manner, by some of the leading citizens of Dundee, who, associating themselves together in a body which they have denominated the Dundee Social Union, have set themselves diligently to work to ascertain all the facts which may help to throw light upon the physical condition and development of the children of the city, and thus to pave the way, by the attainment of actual knowledge, for the introduction of any procedures which may be required for the purpose of mitigating or removing influences unfavourable to the future population. As one step in the general inquiry to be instituted, five elementary schools were selected in different parts of the city. From these five schools a thousand children were taken at random, by calling forward every other, or every third, or every fourth child on the register in the order of their names, and those so taken were carefully weighed, measured, and examined by medical practitioners. The whole inspection was placed under the general direction of the local medical officer of health, and the several examiners worked in concert under a well considered system. The principal facts ascertained up to the present time have been published in an interim report.

From this interim report we do not at present intend to quote further than to say that it discloses among the children examined very large percentages of diseased conditions and of their consequences, of rachitic deformities, of height and weight below all accepted averages, of defective sight and hearing, of carious teeth, and of children dirty and verminous from parental neglect. Dundee has a population of about 170,000, and it would be unfair, in the absence of more extended knowledge, to take a single thousand of the children as fair specimens of its juvenile community. It is, nevertheless, noteworthy that many of the assigned defects are of such a nature that their consequences might be obviated by the exercise of reasonable care. A very large proportion of the cases of defective hearing are said to be at a stage admitting of successful treatment, and a large proportion of the cases of defective sight are attributed to errors of refraction which the use of spectacles would relieve. Carious teeth are largely due to improper feeding, coupled, of course, with total neglect of cleanliness; and bodily filth was mainly a consequence of maternal misconduct or neglect. Many of the children, for example, are described as having been sewn up in their clothes and unable to remove them, and the interim report somewhat naively declares it to be a "hardship" that comparatively clean and well-tended children should be compelled to mix with the dirty and the verminous. Such complaints are no doubt well founded in some instances, although we may be inclined to hope that the impression which they would make upon the examiners has led to some pardonable exaggeration of their frequency, or of the prevalence of the conditions which would produce them. The value of the report, and the sense in which the action of Dundee deserves to be accepted as an example by every densely-populated locality in the kingdom, is derived from the manifest determination of the "Social Union" to get to the bottom of things, and to acquire a power of dealing with facts instead of with vague generalities. The "measurements" obtained at Dundee will not attain their full value until we are able to compare them with corresponding measurements, taken not only in other great industrial centres but also in rural communities, and calculated in their entirety, to display the actual state, and the physical and sanitary prospects of the children of the United Kingdom. Upon this state, and upon these prospects, our future as a governing race must depend; and the magnitude of the stake at issue is such that the questions concerned might not unreasonably be expected to appeal even to Parliament or to society.

